# Prevalence and factors of compassion fatigue among nurse in China: A protocol for systematic review and meta-analysis

**DOI:** 10.1097/MD.0000000000024289

**Published:** 2021-01-22

**Authors:** Man Jin, Jialin Wang, Li Zeng, Wanqing Xie, Ping Tang, Zhongqing Yuan

**Affiliations:** School of Nursing, Chengdu University of Traditional Chinese Medicine, Chengdu, Sichuan, China.

**Keywords:** Chinese nurses, compassion fatigue, factors, meta-analysis, prevalence, protocol

## Abstract

**Background:**

Compassion fatigue is defined as a detrimental consequence of experiencing work-related stress among nurses, which can affect the job performance and harm emotional and physical health. The high risk of compassion fatigue among nurses may be even more severe in China. Although several studies have explored the prevalence and factors of compassion fatigue among Chinese nurses, most data derived merely from the specialty units of the hospital or limited samples, and there is a large heterogeneity among studies. Thus, it is indispensable to systematically summarize the risk factors and prevalence of compassion fatigue among clinical nurse in China.

**Methods:**

Two reviewers will independently conduct comprehensively searches in 9 electronic databases including PubMed, The Cochrane Library, Cumulative Index to Nursing and Allied Health Literature (CINAHL), EMBASE, Web of science, MEDLINE, China National Knowledge Infrastructure (CNKI), WanFang and Chinese Biological Medical Database (CBM) with no search date restriction. Cross-sectional and prospective cohort studies that described the prevalence and factors of Chinese nurses compassion fatigue will be eligible for inclusion. The risk of bias and methodological quality of individual study will be assessed using an adapted quality assessment tool from the Agency for Healthcare Research and Quality (AHRQ). Stata 16.0 software will be used for meta-analysis.

**Results:**

The primary outcome will be the prevalence of 3 dimension of compassion fatigue in Chinese nurses. The secondary outcomes will be comparisons of compassion fatigue scores among Chinese nurse of different education background, marital status, employment forms and professional titles.

**Conclusion:**

This overview will contribute to reveal the prevalence and influencing factors in compassion fatigue among Chinese nurses and provide a scientific evidence for the prediction and prevention in compassion fatigue.

**Registration number:**

The registration DOI is 10.17605/OSF.IO/V34X6.

## 1 Introduction

Compassion fatigue is a common phenomenon among a variety of medical professionals, including nurses. Nurses become the largest group of health care providers with the aim of supporting patients empathetically in accordance with their crucial physical, mental, emotional and spiritual needs while other professionals rarely do.^[1,2]^ As a result, they face more work-related stress. Compassion fatigue is defined as emotional exhaustion owing to prolonged, continuous, and close interaction with traumatizing patients and families, exposure to stress and use of self, which could be considered as “cost of caring”.^[3–5]^ According to Coetzee and Klopper,^[6]^ compassion fatigue refers to the fact that nurses expend more compassion energy than they need to recover, and thus the power to recover is lost. Based on a widespread conceptual model, the term “professional quality of life” (ProQOL) can be used to evaluate compassion fatigue which conceptualized as both positive (compassion satisfaction) and negative (burnout and secondary traumatic stress) aspects.^[4,5,7]^ Burnout and secondary traumatic stress together contribute to increasing the risk of compassion fatigue. It can reveal the comprehensive quality of caring work that clinic nurses experience.

It is recognized that the high risk of compassion fatigue among nurses can lead to numerous negative physical, psychological and behavioral symptoms, including sleep disturbance, depression, professional helplessness, substance abuse and strained personal relationships etc.^[8–10]^ In addition, compassion fatigue not only has detrimental consequence for nurses personal quality of life but also cause a wide range of work-related problems that can affect job performance and have a huge negative impact on their organization.^[2]^ A cross-sectional study from a large urban trauma centers in the United States proved that compassion fatigue may contribute to a decline in patient satisfaction as well as an increased in turnover of nurses and medication errors.^[11]^ Furthermore, it is commonly reported that a decline in quality of medical care, increased financial burden and even the occurrence of medical dispute are associated with CF.

In recent years, numerous researches have reported high prevalence of compassion fatigue in clinic nurse which has received increasing attention. It was estimated that the prevalence of compassion fatigue among healthcare workers ranges from 21.6% to 44.8% depending on the hospital unit or area to which they are assigned, such as emergency oncology, and other inpatient specialties.^[12,13]^ The high risk of compassion fatigue among nurses may be even more striking in China. Previous study revealed that Chinese psychiatric nurses from different provinces generally suffer from severe compassion fatigue and burnout.^[14]^ Lu investigated 165 nursing staff of general hospital, and result showed that the prevalence of moderate to high levels of compassion fatigue was 81.8%, as well as age and perceived stress level were considered as significant predictors of compassion fatigue.^[15]^ Moreover, another survey of 1044 nursing staff from 11 tertiary hospitals in China found that the average levels of burnout and secondary traumatic stress were 81.4% and 80.6%, respectively, and reported that the level of compassion fatigue was associated with marital status, exercise, smoking and work hours per day.^[16]^ Therefore, it is crucial to further clarify the status and influencing factors of compassion fatigue among clinical nurses in China.

Chinese nurses may be vulnerable to compassion fatigue not only because of the serious situation of shortage of clinical nurses in China,^[17]^ which can lead to an increase in work-related stress, but also owing to the greater demand for service quality, the patient-centered holistic nursing model reform and health care reform.^[18]^ As there is greatly difference of the healthcare delivery system between China and Western countries, and additionally, although several studies have explored the prevalence and factors of compassion fatigue among various specialty units of the hospital, the results presented by various studies are not completely consistent and even in contradiction.^[16,19–21]^ Thus, the purpose of the present study was to conduct a systematic review and meta-analysis to provide a comprehensive evaluation of current empirical evidence for the prevalence and factors of compassion fatigue among Chinese clinic nurses.

## 2 Method

### 2.1 Protocol registration

The systematic review and meta-analysis has been registered on the OSF website. The registration DOI is 10.17605/OSF.IO/V34X6 (https://osf.io/v34x6).

### 2.2 Search strategy

We will be systematically searching the electronic bibliographic databases including PubMed, The Cochrane Library, Cumulative Index to Nursing and Allied Health Literature (CINAHL), EMBASE, Web of science, MEDLINE, China National Knowledge Infrastructure (CNKI), WanFang and Chinese Biological Medical Database (CBM) with no search date restriction. Moreover, references to relevant review articles will be manually searched to identify additional studies. The following search terms will be used: compassion fatigue, secondary traumatic stress, vicarious trauma, Professional Quality of Life, ProQOL, nurse and so on (The search terms and strategy that will be used for PubMed are showed in Table 1).

**Table 1:** The search strategy in PubMed.


#1 Compassion Fatigue [MeSH]#2 Compassion Fatigue OR Fatigue, Compassion OR Empathy Fatigue OR Secondary Traumatic Stress OR Secondary Traumatization OR Traumatic Stress, Secondary OR Traumatizations, Secondary OR Vicarious Traumatization OR Vicarious Trauma OR Trauma, Secondary OR Traumatization, Vicarious#3 #1 OR #2#4 Professional Quality of Life OR ProQOL#5 #3 OR #4#6 Nurse [MeSH]#7 Nurse OR Chinese Nurse OR Healthcare#8 #6 OR #7#9 #5 AND #8

### 2.3 Inclusion and exclusion criteria

The inclusion criteria in this systematic review and meta-analysis are as follows:

1. The study design is cohort or cross-sectional study.
2. The population are the registered clinical nurses in China without restriction on the gender.
3. The Professional Quality of Life Scale (ProQOL) was used as instruments to evaluate compassion fatigue.
4. The studies clearly reported the prevalence and influencing factors of compassion fatigue among Chinese nurse.

The exclusion criteria are as follows:

1. The research objects include practice nurses and clinicians.
2. Non-Chinese and English literature.
3. Incomplete or missing research data.
4. Types of studies are editorials, commentaries, protocols, meeting abstracts, and other reviews.

### 2.4 Outcome

The primary outcome will be the prevalence of compassion fatigue, the specific prevalence of compassion fatigue in different regions in China. The secondary outcomes will be comparisons of compassion fatigue scores among Chinese nurse of different education background, marital status, employment forms and professional titles.

### 2.5 Study selection

**Figure 1:**
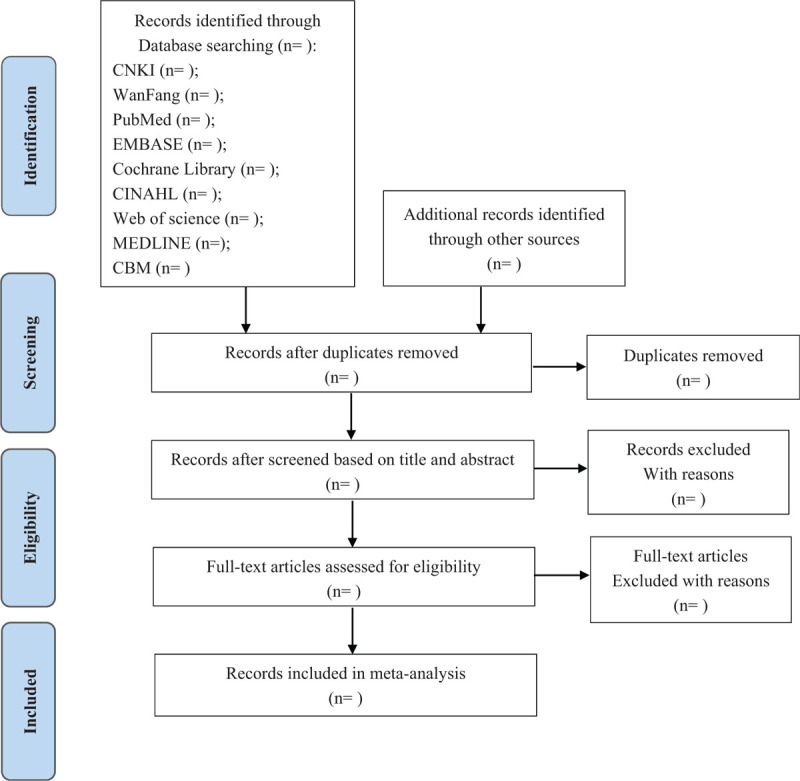
The flowchart of the screening process. CBM = Chinese Biological Medical Database, CINAHL = Cumulative Index to Nursing and Allied Health Literature, CNKI = China National Knowledge Infrastructure.

Papers and records will be identified through inputting search terms (as outlined above) into electronic bibliographic databases. Records will be managed using Endnote X7. Duplicate articles are then deleted from the obtained records. Titles/abstracts were independently screened by the first author and another reviewer (JM and ZL). The full text of these potentially eligible studies will be retrieved and independently assessed for eligibility by 2 review team members. Any significant disagreement identified by the first author and second reviewer will be resolved through consulting with a third reviewer (XWQ). Detailed records will be kept of the number of excluded studies and the reasons for the exclusion, such as: irrelevant to the research topic, without quantitative data and Language is not Chinese or English. To illustrate the selection process of the study, we will also follow the guidelines of Preferred Reporting Items for Systematic Reviews and Meta-Analyses (PRISMA) to form a flow process chart.^[22]^ (Fig. 1.)

### 2.6 Data extraction

A standardized form will be used to extract data from the included studies for assessment of study quality and evidence synthesis. Extracted information will include: first author, publication year, study regions, study population, sample size and participant demographics (including any available details of age, gender etc); the prevalence of compassion fatigue; the factors of compassion fatigue; details of the methodology.

### 2.7 Risk of bias assessment and quality of study

A quality assessment of all included studies will be independently undertaken by the first author and second reviewer using an adapted quality assessment tool from the Agency for Healthcare Research and Quality (AHRQ).^[23]^ This tool assesses for risk of bias of up to 11 specific areas of cross-sectional research studies and is suitable for quality evaluation of the papers included in this systematic review. The answer “yes” is 1 point, and “unclear” or “no” is not score. Quality scores for each study will be presented in a table.

### 2.8 Data synthesis

We will use STATA 16.0 software (Stata Corporation, College Station, TX) to perform analysis. The weighted mean difference (WMD) is used as the combined effect size for measurement data. Heterogeneity of studies will be assessed by using the *I*^2^ statistic,^[24]^ which is a quantitative measure of inconsistencies in different studies. We will consider 25% to 50%, 50% to 75%, and >75% as low, moderate, and high heterogeneity, respectively.^[25]^ Fixed or random effects models will be used properly based on the *I*^2^ statistic. If *P* ≥ .1, *I*^2^ < 50%, the fixed effect model is adopted. Otherwise, it is considered that there is higher heterogeneity between the studies, and the random effect model should be used. In addition, we will conduct a sensitivity analysis to test the impact of a single study on the combined estimates of each influencing factor.

### 2.9 Publication bias

When the number of included studies in each outcome is sufficient, Egger test and Begg test will be used to evaluate publication bias.^[26]^ (n > 10)

## 3 Discussion

Compassion fatigue can not only induce a variety of physical and mental diseases, but also lead to a decline in the level of work engagement, resulting in loss of work enthusiasm, low-level work efficiency and even medical disputes and medical negligence. This systematic review and meta-analysis will contribute to reveal the prevalence and influencing factors in compassion fatigue among Chinese nurses. We hope our work could provide a scientific evidence for the prediction and prevention in compassion fatigue, ultimately to improve the quality of health care.

## Author contributions

**Conceptualization:** Man Jin.

**Data curation:** Man Jin, Li Zeng, Wanqing Xie, Ping Tang, Zhongqing Yuan.

**Funding acquisition:** Jialin Wang.

**Methodology:** Man Jin, Li Zeng.

**Software:** Man Jin, Wanqing Xie.

**Writing – original draft:** Man Jin.

**Writing – review & editing:** Man Jin, Jialin Wang, Li Zeng.
